# BUILDing SCHOLARS: A program exemplar at a Hispanic serving institution to develop biomedical researchers

**DOI:** 10.1371/journal.pone.0315298

**Published:** 2024-12-30

**Authors:** Rafael Aguilera, Guadalupe Corral, Angelica Monarrez, Amy E. Wagler, Lourdes E. Echegoyen

**Affiliations:** 1 Research Evaluation and Assessment Services, The University of Texas at El Paso, El Paso, Texas, United States of America; 2 Department of Public Health Sciences, The University of Texas at El Paso, El Paso, Texas, United States of America; 3 Campus Office of Undergraduate Research Initiatives, The University of Texas at El Paso, El Paso, Texas, United States of America; 4 Department of Chemistry and Biochemistry, The University of Texas at El Paso, El Paso, TX, United States of America; Medical College of Wisconsin - Central Wisconsin Campus, UNITED STATES OF AMERICA

## Abstract

The student development initiatives of the BUILDing SCHOLARS Center at The University of Texas at El Paso comprise a program intended to prepare undergraduate students to enter and succeed in advanced graduate and professional biomedical degree programs, ultimately contributing to the diversity of the biomedical research workforce. The program adopted the Johnson/Bozemann Asset Bundles model, which recommends addressing five areas necessary to support minority students as they prepare for and continue towards scientific careers: a) educational endowments, b) science socialization, c) network development, d) family expectations and e) material resources. Through a variety of activities, which included a minimum of two years of research training, all five asset bundles were integrated into the program. Validated scales on science identity and research self-efficacy were completed by program fellows, and academic metrics such as retention, grade point average (GPA), and time to degree were collected for both program fellows and a comparison group. Thorough records of all students who participated in the program, including their presentations and co-authored publications, have been maintained, and students are being tracked post-graduation to determine their entry and completion of advanced degrees. Academic-based results for the first three cohorts of program participants show large, significant, and positive differences in retention, 4-year graduation rates and entry into advanced degrees between program participants and the comparison group. Comparison of academic and non-academic metrics indicate that the asset bundles approach is effective in retaining and preparing students for advanced degrees and careers in STEM disciplines. Using our results in combination with research by others on retention of students in STEM, we suggest factors contributing to the push-out of very talented majority Hispanic students in the comparison group from completing STEM degrees or not pursuing advanced studies in STEM areas.

## 1. Introduction

There are multiple benefits associated with inclusion of individuals from a diversity of backgrounds in the biomedical research workforce (broadly defined here to include clinical, social, and behavioral sciences). In addition to fostering scientific innovation [1–4], a diverse biomedical workforce can ensure that underserved and populations that experience health disparities not only participate in health research as investigators and as subjects, but also benefit from its outcomes [5]. However, evidence gathered through multiple studies in the past few years indicates that there continues to be a significant underrepresentation of minoritized groups in the general science, technology, engineering, and mathematics (STEM) workforce. As of 2021, the percentage of Hispanics and Blacks in the U.S. population was 18.5 and 13.4, respectively, while their representation in the STEM workforce were only 8% and 9%, respectively [6]. Many factors contribute to this disparity. Although an increase in the number of Ph.D. degrees in the biomedical sciences earned by individuals from traditionally underrepresented backgrounds has been observed [7], the attrition rate of scientists from these underrepresented groups continues to be an issue [8]. Lack of role models at the professoriate level is one of many limiting factors [9]. The lack of role models has been linked with the racial/ethnic disparity in funded awards from the National Institutes of Health (NIH) [10,11], the origins of which may be associated with systemic issues such as implicit and other biases [12]. As a result of these and other studies, the NIH put in motion the Diversity Initiative in 2012, in an attempt “to fundamentally shift the way scientists are trained and mentored to attract and retain individuals from underrepresented groups in the scientific workforce.” [13] “The diversity initiative is a national collaborative through which the Diversity Program Consortium (DPC), in partnership with the National Institutes of Health (NIH), was commissioned to develop, implement, and determine the effectiveness of approaches to strengthen institutional capacity to engage individuals from diverse backgrounds and help them prepare for and succeed in biomedical research careers. The DPC is providing a unique opportunity to understand and address multi-dimensional factors (at the institutional, social, and individual levels) that may strongly influence student success, professional development, and persistence within biomedical research career paths” [13].

Funded by the NIH through their Common Fund, and as part of their Diversity Initiative, BUILDing SCHOLARS (Building Infrastructure Leading to Diversity–Southwest Consortium of Health Oriented education Leaders and Research Scholars) started as a Center of Excellence at the University of Texas at El Paso (UTEP) in the fall of 2014. As one of ten BUILD sites across the U.S., the center comprises three sets of initiatives designed to address some challenges faced by students, faculty, and the institution in the process of advancing diversity in the biomedical research workforce. Established at an institution with a student population that ranges between 82% and 87% Hispanic, the ultimate goal of the center is to develop the next generation of biomedical researchers from the Southwest region of the United States and to contribute to a substantial increase in the diversity of the NIH funded workforce at the national level. Although student development is intimately related to the project’s faculty and institutional development initiatives, this article focuses exclusively on the student development aspects of the program and their impact. Results of the other two sets of initiatives will be discussed in other reports and publications.

Of note, the ten BUILD sites across the U.S. along with the National Research Mentoring Network (NRMN) and the Coordination and Evaluation Center (CEC), comprise the NIH Diversity Program Consortium (DPC), a network of institutions, funded by NIH, to improve training and mentoring and to enhance individuals’ success in biomedical research careers [13].

### 1.1. BUILDing SCHOLARS student development program rationale

For the BUILDing SCHOLARS student development initiatives (from here on referred to as “the program”), the specific objectives are to broaden the pool of talented participants, improve their retention in biomedical science majors and increase acceptance and entry into competitive graduate and advanced professional biomedical degree programs. With this in mind, the principal investigators adopted the Johnson and Bozemann *Asset Bundles Approach* to training [14]. This approach combines the scientific and technical human capital theoretical framework with the theory of social identity contingencies [15,16] to address areas that are considered critical to support minority students as they prepare for and continue towards scientific careers. The approach recommends that institutions create student-training programs that focus on five types of asset bundles: educational endowments, science socialization, network development, family expectations and material resources [14]. A more detailed description of these asset bundles in connection to all BUILD program activities is presented in section 1.6 below. However, it is important to note that in terms of educational endowments, the program focused on experiential practices known to have high impact on learning and retention of STEM students [17]. We specifically included:

- First year seminars/experiences via participation in a sequence of Course-based Undergraduate Research Experiences (CUREs) offered at the freshman level,
- Traditional (mentored) undergraduate research experiences (UREs),
- Internships, not in the form of an employment practicum but in the form of summer research experiences at research partner institutions,
- Common intellectual experiences through participation in professional development workshops and presentations at local symposia, and
- Capstone experiences in developing an undergraduate thesis.

Details of specific training elements and their connection to each asset bundle are presented in the sections that follow.

Central to contextualizing the importance and impact of such an approach is the demographics of the undergraduate student population at UTEP, which have consistently been, since the program was conceived in 2013, 82–85% Hispanic, 50–52% first generation students, 53–56% female, and 60–63% Pell grant eligible.

### 1.2. Student training

**Fig 1:**
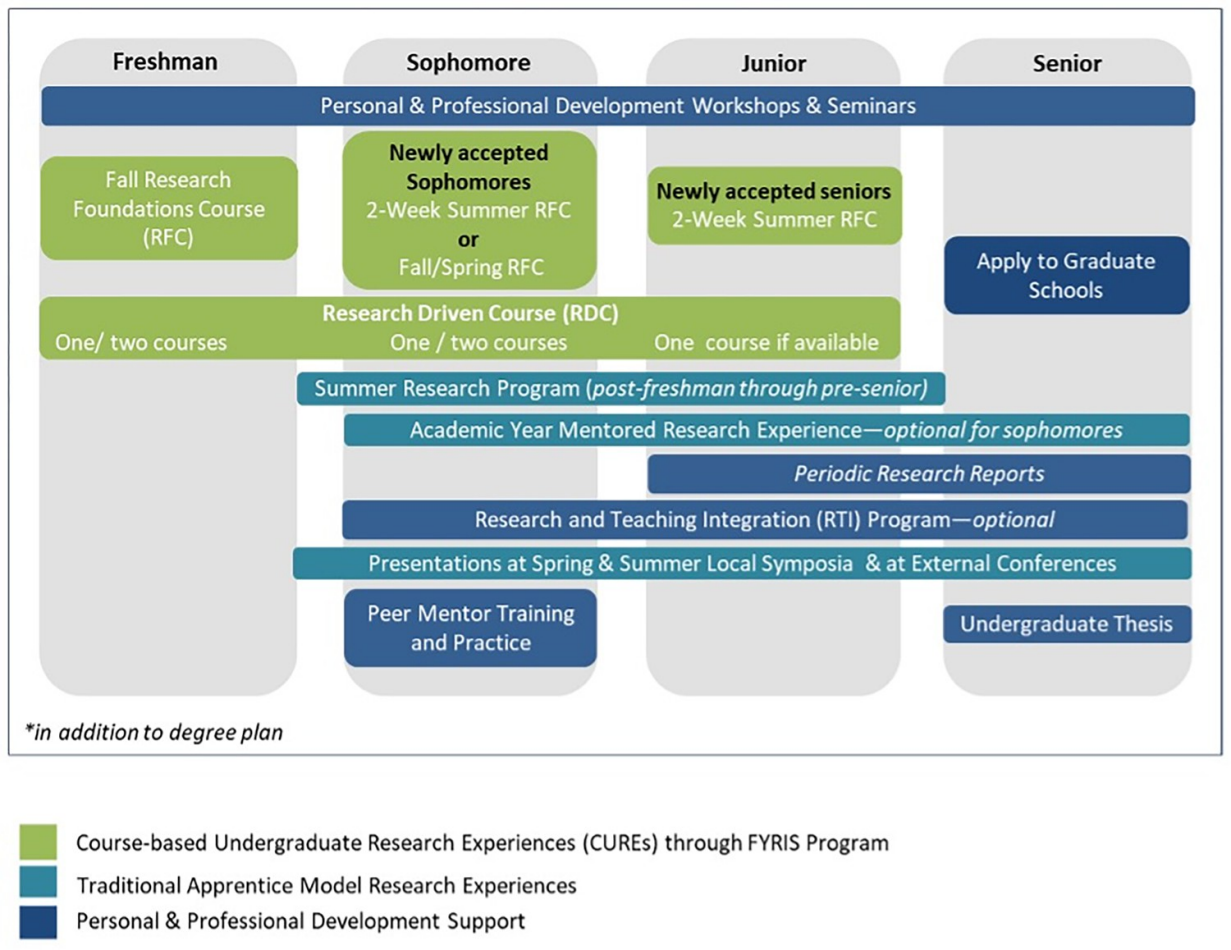
BUILDing SCHOLARS program student training sequence.

The student training sequence shown in Fig 1 is in addition to the required course work that students must complete, according to their degree plan, to graduate with their bachelor’s degree from UTEP.

**Requirements for all program participants:** In order to ensure that students graduated within the time stipulated by the program (e.g. 4 years for entering freshman, 3 years for entering sophomores or 2 years for entering juniors), the program required them to complete 30 credit hours per academic year, with no courses taken in the summer. Each year before graduation, all students were expected to participate in a summer research experience (SRE) at one of our multiple research partner institutions for 10 summer weeks. Requirements for the academic year research are described below for each specific student classification. All students were expected to attend a series of professional development workshops that provided supplementary training needed for participation in research as undergraduates, as well as preparation for graduate school and research careers. Some of these workshops were writing intensive and/or intended to guide students for follow-up writing requirements. Students were assigned to workshops based on their classification and whether or not they had participated in a given workshop in prior years. For a list of professional development workshops and how students were assigned to specific workshops, see Table 1 below.

**Table 1:** BUILDing SCHOLARS professional development workshops.

Research Training	How to be a Good Research Mentee^1^
	How to be a Good Peer Mentor^1^
	The Research Notebook^2^
	Responsible Conduct of Research Basics^2^
	Preparing an Abstract for a Conference Presentation*^2^
	Preparing Research Reports and an Undergraduate Thesis*^2^
	Traveling to and Attending Conferences (incl. applying for funding)^2^
	Responsible Conduct of Research Refresher^3^
Graduate School Preparation	Applying to Graduate School^4^
	The Graduate School Interview^5^
	Crafting a Personal Statement*^1^
	GRE Preparation^5^
Professional Development	Introduction to NIH and NIH Careers^1^
	Finding Work-Family Balance in a Research Career^2^
	Preparing Resumes and CV’s*^1^
	Entrepreneurship^3^
Summer Preparation	Summer Program at Partner Institutions: Application Process^1^
	Summer Program Pre-departure Orientation^1^

* Writing Intensive; Attendance requirements:

^1^All incoming students

^2^All students participating in research

^3^Seniors

^4^Sophomores and Juniors

^5^Juniors.

In service of the primary goal, one major objective of the program was to prepare students for entry into and success in advanced degree programs in the biomedical sciences. For this reason, the program required students to maintain a GPA of 3.3 or above, given that this is a common GPA cut-off for many graduate programs.

In addition to all these requirements, given the imperative from NIH to determine the effectiveness of approaches, students were strongly encouraged to complete all program evaluation surveys coming from the UTEP site program evaluator as well as those from the Coordination and Evaluation Center (CEC). The CEC was also a NIH grantee site responsible for the longitudinal, consortium-wide evaluation of the training and mentoring interventions that all BUILD and the NRMN awardees develop and put into practice.

### 1.3. Selection and program welcome process

For the purpose of the program, the NIH definition of Biomedical Sciences, which includes the natural sciences as well as social and behavioral sciences and biomedical engineering, is used [18]. Students were accepted to the program as entering freshman, rising sophomores, or rising juniors (no more than 60 credit-hours), with the goal of getting students to participate in the program for a minimum of two whole academic years. The two-year minimum participation is based on studies indicating that participation in undergraduate research for 2-years increases the likelihood of application to graduate programs and provides students with the mentoring and readiness necessary to succeed [19].

To apply to the program, students completed an online application that requested academic and personal information, as well as a transcript and personal statement.

Each application was blindly reviewed (name, gender, race/ethnicity are redacted) and scored by three members of the BUILD leadership team using a rubric. Scores were normalized and, after a thorough discussion of applicants, a group of top candidates was pre-selected and invited to an interview conducted by two randomly assigned members of the leadership team. Scores from the application and interview were combined and the team met once again to discuss finalists and made acceptance decisions. Students were selected based on their potential to complete the program and likelihood to pursue a graduate degree and a career as a biomedical researcher. Given the student demographics at UTEP and the goal of NIH to enhance diversity in the biomedical research workforce, the principal investigators agreed that the blind selection process would ensure minimal bias and achieve the diversity target, which is, at a minimum, to mirror the demographics of the university. The latter has not only been the case during the program history, including the cohorts that comprise this study, but it has also been surpassed, with higher levels of underrepresented students than reflected at the university. Table 2 shows that for the first three cohorts, Hispanic/Latinos represented 93.6% of program participants.

**Table 2:** Full cohort demographics 2015–16 through 2017–18*.

	2015–16 Cohort		2016–17 Cohort		2017–18 Cohort		Total	
	N	%	N	%	N	%	N	%
**Total**	53	100%	85	100%	98	100%	236	100%
**Hispanic or Latino**	49	92.45%	80	94.12%	92	93.88%	221	93.64%
**White (non-Hispanic)**	2	3.77%	3	3.53%	5	5.10%	10	4.24%
**Black (non-Hispanic)**	0	0.00%	0	0.00%	1	1.02%	1	0.42%
**Asian**	1	1.89%	1	1.18%	0	0.00%	2	0.85%
**Native American**	0	0.00%	1	1.18%	0	0.00%	1	0.42%
**Other**	0	0.00%	0	0.00%	0	0.00%	0	0.00%
**Not reported**	1	1.89%	0	0.00%	0	0.00%	1	0.42%

*Not unique students in all cohorts, as some students in subsequent cohorts were continuing.

As soon as students were officially accepted, they, together with their parents, were invited to two events. The first event was an orientation where the terms of the scholarship agreement and a program overview and expectations were presented and discussed. In addition, students were introduced to the program’s dedicated academic advisor. The second event combined a welcome ceremony for incoming students with a graduation ceremony for graduating seniors. This second event was attended by multiple members of the university senior leadership (President, Vice-Presidents, and Deans), which gave parents and students a sense of how important this program is to the university and the nation. At the event, two panels of faculty and former students provided perspectives on the benefits and challenges of program participation, and a workshop for the parents helped them understand how they could support their students as they go through the program’s rigorous training.

### 1.4. Research nodes

To optimally match students with top research mentors and their corresponding projects in biomedical research, the principal investigators initially conducted an analysis of the research areas where UTEP demonstrated significant strength, not only in terms of funding and publications but also in the availability of outstanding research mentors willing to accept undergraduate researchers. Furthermore, research partner institutions were selected based on their willingness to collaborate with the BUILDing SCHOLARS center on several initiatives, including their capacity to accept program fellows for summer research experiences into research groups where these areas are emphasized. The analysis resulted in seven research nodes having multiple mentors demonstrating the above characteristics. Therefore, students accepted into the program had to demonstrate an interest in engaging in research in one or more of these areas both with a UTEP mentor during the academic year and with a mentor at a research partner institution each summer. These areas are listed in Table 3, along with the number of students whose research focused on each area from the three cohorts presented here. Students were allowed to select a summer project in an area different from the one they focused on during the academic year research. However, unless extenuating circumstances ensued, they were encouraged to stay with the same project/mentor during the last two academic years to produce a more comprehensive thesis.

**Table 3:** Research nodes.

Addiction
Cancer
Infectious diseases
Neurodegenerative diseases
Environmental health
Health disparities
Translational biomedicine

### 1.5. Program benefits

Listed below are specific benefits that the program provided to its fellows. All benefits are discussed in more detail in various sections throughout this article. These benefits are mapped to specific Asset Bundles in Table 4, section 1.6.

**Table 4:** BUILDing SCHOLARS student development activities mapped to type of asset bundle.

Activities		Asset Bundle*					Student Classification
		EE	SS	ND	FE	MR	
**Research Training Activities**	**Completion of FYRIS program CUREs**	•	**•**			**•**	FR
	**Traditional Academic Year Research Experience**	**•**	**•**	**•**		**•**	JR & SR mandatory
	**Summer Research Experience at a Partner Institution**	**•**	**•**	**•**		**•**	FR & SO optional
	**Research Report Writing (coaching from program science writer)**	**•**	**•**				JR & SR
	**Thesis Writing (with coaching from program science writer)**	**•**	**•**				SR
	**Research Presentations at Local Conferences**	**•**	**•**	**•**	**•**	**•**	SO, JR, SR
	**Research Presentations at Regional & National Conferences**	**•**	**•**	**•**	**•**	**•**	SO, JR, SR
	**Guest Speaker Seminars**	**•**	**•**	**•**	**•**	**•**	All
**Academic Support**	**Personalized degree plan advising**	**•**					All
	**Tutoring & Supplemental Instruction**	**•**				**•**	All
Workshops & Events	**Professional Development Workshops**	**•**	**•**	**•**			All
	**GRE training**	**•**	**•**			**•**	JR
	**Program Welcome Event**	**•**	**•**	**•**	**•**		All
	**Workshop for Parent(s) of Incoming Students**	**•**			**•**		FR, SO, JR
	**Biomedical Research Careers Workshop/Panel (parents invited)**	**•**	**•**	**•**	**•**		All
	**Summer Research Program Pre-departure Orientation**	**•**	**•**		**•**		FR, SO, JR
	**BUILDing SCHOLARS Graduation Ceremony**	**•**	**•**		**•**		All
**Other Mentoring Activities**	**Peer-mentor training**	**•**	**•**	**•**			SO, JR
	**Social Gatherings with PIs & Mentors**	**•**	**•**	**•**	**•**		All
	**First year informal mentoring by a PI**	**•**	**•**	**•**			FR
**Direct Funds**	**Tuition Scholarship & Monthly Stipend**					**•**	All
	**Research Materials and Supplies (AY and Summer)**					**•**	All
	**Publication Fees**					**•**	All

*EE = Educational Endowment; SS = Science Socialization; ND = Network Development; FE = Family Expectations; Material Resources.

#### 1.5.1 Financial

- 60% tuition for 30 credit hours per year
- Monthly stipend for 12 months/year based on yearly updated NIH Ruth Kirschstein guidelines [20]
- Registration, travel and accommodations to present research results at external regional or national conferences
- Materials and supplies yearly allotment to cover basic research expenditures
- Travel, housing, and meals to participate in a summer research experience (SRE) at one of the research partner institutions.

#### 1.5.2 Advising and mentoring

- Personalized academic advising by a trained program staff
- First year mentoring by a member of the program leadership
- Assistance in selecting an academic year research mentor
- Assistance in selection of an SRE program/institution
- First year peer-mentoring

#### 1.5.3 Academic

- Tutoring support when unavailable at the university tutoring center
- Priority registration for courses
- Participation in CUREs as freshman
- Participation in research under the mentorship of a UTEP faculty member during the academic year and a research partner institution faculty member during at least 10 summer weeks.

#### 1.5.4 Professional development

- Engagement in professional development workshops (see Table 1 above)
- Writing coach support by the program science writer (personal statements, CV/resume, research reports, thesis)

**Juniors and seniors** As part of their training, students submitted a research project proposal during the first month of their junior year, which they prepared in collaboration with their faculty research mentor. After the proposal was accepted by the science writer and approved by leaders of the student training core of the program, students continued their projects and submitted periodic research reports, approximately every six weeks. The reports were in the format of a journal article of the mentor’s choosing and were reviewed and approved by the mentor before submission to the program science writer. The science writer reviewed the reports and provided feedback to students, with the idea that the last report in the fall semester of the senior year was converted into an undergraduate thesis and submitted before graduation. In addition, during the senior year, students were highly encouraged to submit a minimum of three completed graduate school applications.

**Sophomores** participated in peer-mentor training and were assigned to help an incoming freshman in the process of navigating the university and program requirements; this activity also served to build community with other program fellows. Sophomores were not required to participate in academic year research but were strongly encouraged, and many of them elected to do so after participation in summer research at partner institutions and in the Freshman Year Research Intensive Sequence (FYRIS) program.

**Incoming freshmen** were expected to enroll in a 3-course sequence of Course-based Undergraduate Research Experiences (CUREs) established via the Freshman Year Research Intensive Sequence (FYRIS) program at UTEP. For a full FYRIS program description see [21]. The sequence provided early exposure to research methods through a research foundations course and to faculty developed authentic research projects through laboratory-based courses with different research themes in the Biological Sciences, Chemistry/Biochemistry and Geology. Program fellows were also assigned a first-year faculty mentor, one of the eight Principal Investigators (PIs), with whom they met monthly throughout their first year. The idea was for both student and mentor to get to know each other, for the student to provide feedback on their progress, discuss any issues (academic or personal) they may have needed advice on, and receive extra guidance on navigating the university and/or courses. Additionally, incoming freshmen were assigned a peer mentor, a sophomore or junior in the program who went through peer-mentor training and had one or two years of experience in the program. The peer mentors helped the freshman mentees navigate university resources while developing a sense of community. In the summer, freshmen (like all other program participants) were sent to a research partner institution of their choice to conduct research.

### 1.6. Asset bundles

As indicated in section 1.1, the program adopted the Johnson and Bozemann *Asset Bundles Approach* to student training. Johnson et al., defines “asset bundles” as the “specific sets of abilities and resources individuals develop to help them succeed educationally and professionally”. In that context, the approach recommends that institutions create student-training programs that focus on following five types of asset bundles [14]:

**Educational endowments (EE)**: innovative courses, resources and educational support to strengthen existing assets and build upon those. These endowments must mitigate potential deficits and strengthen students’ math and science skills.

**Science socialization (SS)**: activities that enable students to form a personal identity compatible with a possible career in academic medicine or STEM profession.

**Network development (ND)**: activities that allow students to build positive social capital through mentoring relationships, involvement in extracurricular activities, and peer influence.

**Family expectations (FE)**: activities that encourage or mitigate interpersonal dynamics within families that operate to encourage or discourage children from pursuing higher education and careers in academic medicine and STEM fields.

**Material resources (MR)**: resources that mitigate the financial burden to fund college students of low socio-economic status, who work to support themselves and miss out on a number of opportunities that result in attrition or less competitive preparation.

To demonstrate that the model is fully operational, Table 4 maps out each activity in the program to one or more of the asset bundles.

## 2. Program evaluation

The Diversity Program Consortium (DPC) developed a set of Hallmarks of Success at the Student, Faculty and Institutional levels to guide the Consortium-wide evaluation and determine overall DPC impact [22]. Student Hallmarks are shown in Table 5 below. For clarity, note the inclusion of the data source used for evaluating results on each hallmark. At the individual site level, BUILDing SCHOLARS also used DPC Student Hallmarks to assess the impact the program had on student participants. Given the program’s nature, not all hallmarks needed to be included in our evaluation. For example, *intent to pursue a career in biomedical research (STU-7)* is predetermined to a certain extent during the participant selection process, and *frequent receipt of mentoring to enhance success in the biomedical pathway* (STU-10) and *participation in mentored or supervised biomedical research (STU-11)* are distinguishing features/requirements of our program design, as described in section 1.2 to 1.6 and Table 4 above.

**Table 5:** Enhancing the diversity of the NIH-funded workforce hallmarks of success [22].

Hallmark	Student/ Trainee Hallmarks of Success	Assessment Data Origin
**STU-1**	High academic self-efficacy	GPA^1^
**STU-2**	High self-efficacy as a researcher	Survey^2^
**STU-3**	High science identity	Survey^3^
**STU-4**	Satisfaction with quality of mentorship	Survey^4^
**STU-5**	Perceived sense of belonging within the university	Survey^4^
**STU-6**	Perceived sense of belonging within the research community	Survey^4^
**STU-7**	Intent to pursue a career in biomedical research	Intrinsic^5^
**STU-8**	Entry into an undergraduate biomedical degree program	Intrinsic^5^
**STU-9**	Persistence in biomedical degree or other formal research training program	Retention^1^
**STU-10**	Frequent receipt of mentoring to enhance success in the biomedical pathway	Intrinsic^5^
**STU-11**	Participation in mentored or supervised biomedical research	Intrinsic^5^
**STU-12**	Evidence of competitiveness for transitioning into the next phase in the biomedical career pathway	GPA^1^, Research Reports^6^, Publications^6^, Presentations^6^, Thesis^6^
**STU-13**	Participation in academic or professional organizations related to biomedical disciplines	Not collected
**STU-14**	Evidence of excelling in biomedical research and scholarship	Publications, Presentations, Thesis
**STU-15**	Strong academic and professional networks	Intrinsic^5^
**STU-16**	Completion of biomedical degree or other formal training program	Graduation^1^
**STU-17**	Application and acceptance to a subsequent research training program in a biomedical discipline	Database^6^ & Student Clearinghouse^1^
**STU-18**	Entrance into a subsequent research training program in a biomedical discipline	Database^6^ & Student Clearinghouse^1^

^1^UTEP Center for Institutional Evaluaton, Research and Planning.

^2^Adapted from Bieschke et al. [23].

^3^Adapted from Chemers et al. [24].

^4^Not included in this study.

^5^From program application data.

^6^BUILDing SCHOLARS student database.

## 3. Methodology

The present research incorporates two types of data and two separate groups of program cohorts. For academic metrics, we focused on the program’s first three cohorts, 2015, 2016, and 2017, as those students graduated (4-year graduation rate) up to the end of Spring 2021. For non-academic metrics, we rely on survey data obtained from annual research experience surveys administered to the program fellows engaged in the mentored research experience between the 2019–2020 and 2021–2022 academic years. Each annual research experience survey was administered at the end of the semester in May, one week prior to finals and two weeks after, for a total of three weeks. Specifically, the 2019–2020 data were obtained May 6–22, 2020, the 2020–2021 data were collected May 3–21, 2021, and the 2021–2022 data were obtained May 2–20, 2022. We selected survey data from those years because the hallmarks of success (Table 5), including research self-efficacy (STU-1) and science identity (STU-2), were announced by the DPC in 2019; therefore, the validated scales measuring these hallmarks were first used on the annual research experience surveys at the end of the 2019–2020 academic year. It is important to note, however, that the fellows in the cohorts who completed the annual research experience survey assessing research self-efficacy and science identity took part in the same program activities as the fellows in all preceding cohorts. Data collection and metrics are explained in further detail below. This study was conducted with IRB approval (#1462758)

### 3.1 Academic metrics: Retention, graduation, GPA, publications, and post-graduate enrollment

We obtained BUILD fellow data and comparison group aggregate data directly from institutional student records to assess academic outcomes such as retention, time to degree and GPA. Students’ co-authored publications were tracked using the NIH MyNCBI system, and post-graduate enrollment was obtained directly from students’ responses to our direct inquiries and verified using the National Student Clearinghouse.

To assess the success of the program, students in the program were compared to students not in the program with similar academic characteristics. First, we only included 108 students in the study, out of the 236 program participants in the 2015, 2016 and 2017 cohorts. Only those who started at UTEP as first-time freshman (FTF), even if they started participating in the program as sophomores or juniors were included. See Table 6 for study participant cohort size and demographics. Second, we determined that the average GPA for those 108 students in the program at the end of their freshman year was 3.7. Third, we determined the majors of the 108 students in the three program cohorts. For a fair comparison, the comparison group consisted of non-program students, who started as FTF the same years as the program fellows, had a 3.7 GPA or higher at the end of their first year and were in the same majors (e.g., biology, psychology) as those in the program. Aggregate data from a total of 1,312 students were obtained and included in the comparison group.

**Table 6:** Study participant demographics*.

	2015–16 Cohort		2016–17 Cohort		2017–18 Cohort		Total	
	N	%	N	%	N	%	N	%
**Total**	51	100%	37	100%	20	100%	108	100%
**Hispanic or Latino (all races)**	47	92.20%	35	94.59%	17	85.00%	99	91.67%
**White (non-Hispanic)**	3	5.80%	2	5.41 %	0	0.00%	5	4.62%
**Black (non-Hispanic)**	0	0.00%	0	0.00%	0	0.00%	0	0.00%
**Asian**	1	2.00%	1	0.00%	0	0.00%	2	1.85%
**Native American**	0	0.00%	1	0.00%	0	0.00%	1	0.93%
**Two or more races**	0	0.00%	0	0.00%	2	10.00%	2	1.85%
**Other**	0	0.00%	0	0.00%	0	0.00%	0	0.00%
**Not reported**	0	0.00%	0	0.00%	1	5.00%	1	0.93%

*Not unique students in all cohorts, as some students in subsequent cohorts were continuing.

### 3.2. Non-academic metrics: Research self-efficacy, science identity and quality of the research experience

Non-academic metrics, in particular research self-efficacy and science identity, have long been identified by scholars as important psychosocial constructs necessary for undergraduates’ developmental process as they navigate their undergraduate research careers [23–25]. These two metrics correspond to hallmarks STU-1 and STU-2 (Table 5). Unlike the previous section, where we examined academic metrics of cohorts 2015, 2016, and 2017 (due to those cohorts having completed at least 4 years since they began), here we examine cohorts that participated in our Academic Year–Mentored Research Experience surveys between the 2018–2019 and 2021–2022 academic years, including validated scales using retrospective pre-experience and post-experience measures of research self-efficacy [23] and science identity [24] to assess change in hallmarks of success selected by the DPC.

It is also important to note that we do not use survey responses from all classes (i.e., freshman, sophomores). Previous research indicates that participants tend to exaggerate or minimize ratings on behavioral scales the first time they respond to them [26]. Furthermore, development of adolescents’ identities, including science identity, is a complex process that takes several years [27]. Indeed, we observed this phenomenon in our own measures. For this reason, we limit our analyses to only juniors and seniors. Finally, results presented below are cross-sectional by design, rather than longitudinal, due to the lack of power and sample size necessary for valid, longitudinal analyses.

#### 3.2.1 Research self-efficacy

Near the end of every academic year in May, the program evaluation team administered the Academic Year–Mentored Research Survey to all current program fellows. This survey consists of several measures, two of which are research self-efficacy and science identity scales. Research self-efficacy is defined as the extent that someone believes they are capable of carrying out various research tasks [22]. The research self-efficacy scale is adapted from Bieschke, Bishop, and Garcia’s [23] and was modified to include a retrospective measure. That is, participants were asked to indicate their level of disagreement or agreement with positive statements about their ability to conduct research-related activities before and after their academic year research experience. Participants used a 5-point Likert-type scale ranging from 1 = *Strongly Disagree* to 5 = *Strongly Agree* to indicate how confident they were in 18 research-related abilities. A sample item included “I can explain my research problem by drawing the necessary connections with prior research results.” The full scale used is in the supplementary materials.

#### 3.2.2 Science identity

Science identity was measured using a modified, 4-item scale adapted from Chemers, et.al [24]. Science identity refers to the degree to which one identifies as a “scientist” and is comprised of one’s interest in science, their abilities and competencies in science, as well as the extent that others identify one as a scientist. Participants used a scale ranging from 1 = *Strongly Disagree* to 5 = *Strongly Agree* to indicate the extent that each of the four statements was true of them. A sample item includes “I have a strong sense of belonging to the community of scientists”. As with the research self-efficacy scale, the full science identity scale used is included in the supplementary materials. Participants were asked to respond to the four items in reference to before and after the academic year research experience.

#### 3.2.3 Quality of the research experience

The quality of program fellows’ research experiences was measured in three different ways. First, a separate comprehensive study has been conducted using the Mentoring Competency Assessment (MCA) survey [28] (paired mentor-mentee surveys), for which a manuscript is in preparation. Hence a methodology and results will not be included here, as it has all been included in that manuscript.

A second approach assessed each student presentation of their domestic research work at the local spring symposia. Each presentation was judged by invited faculty, graduate students and post-doctoral fellows, using a standardized rubric [29] with each student receiving at least 3 individual scores, which were then normalized.

Third, the quality of the fellows’ summer research experience at partner institutions was also measured using a combination of scores of their presentations at a local end of summer symposium upon their return to campus, a self-assessment of their research experiences survey and the MCA [30].

Finally, the quality of the periodic research reports submitted by students (leading to their undergraduate thesis), was monitored by the program science writer. Each research report, submitted approximately every 6 weeks, had to be approved by the research mentor prior to submission by the student. The science writer provided feedback on the quality of each report for students to incorporate the feedback in subsequent reports. Early in the semester of graduation, the complete thesis had to be submitted for acceptance by the university honors program.

## 4. Results & discussion

Academic data results presented here refer to the program’s 2015, 2016 and 2017 cohorts, as those students graduated (4-year graduation rate) by the end of Spring 2021. For these students we collected academic information about their retention throughout 4 years, GPA at graduation and post-graduate enrollment in either graduate or professional schools.

### 4.1. Academic metrics: Retention, graduation, GPA, publications and post-graduate enrollment

**Fig 2:**
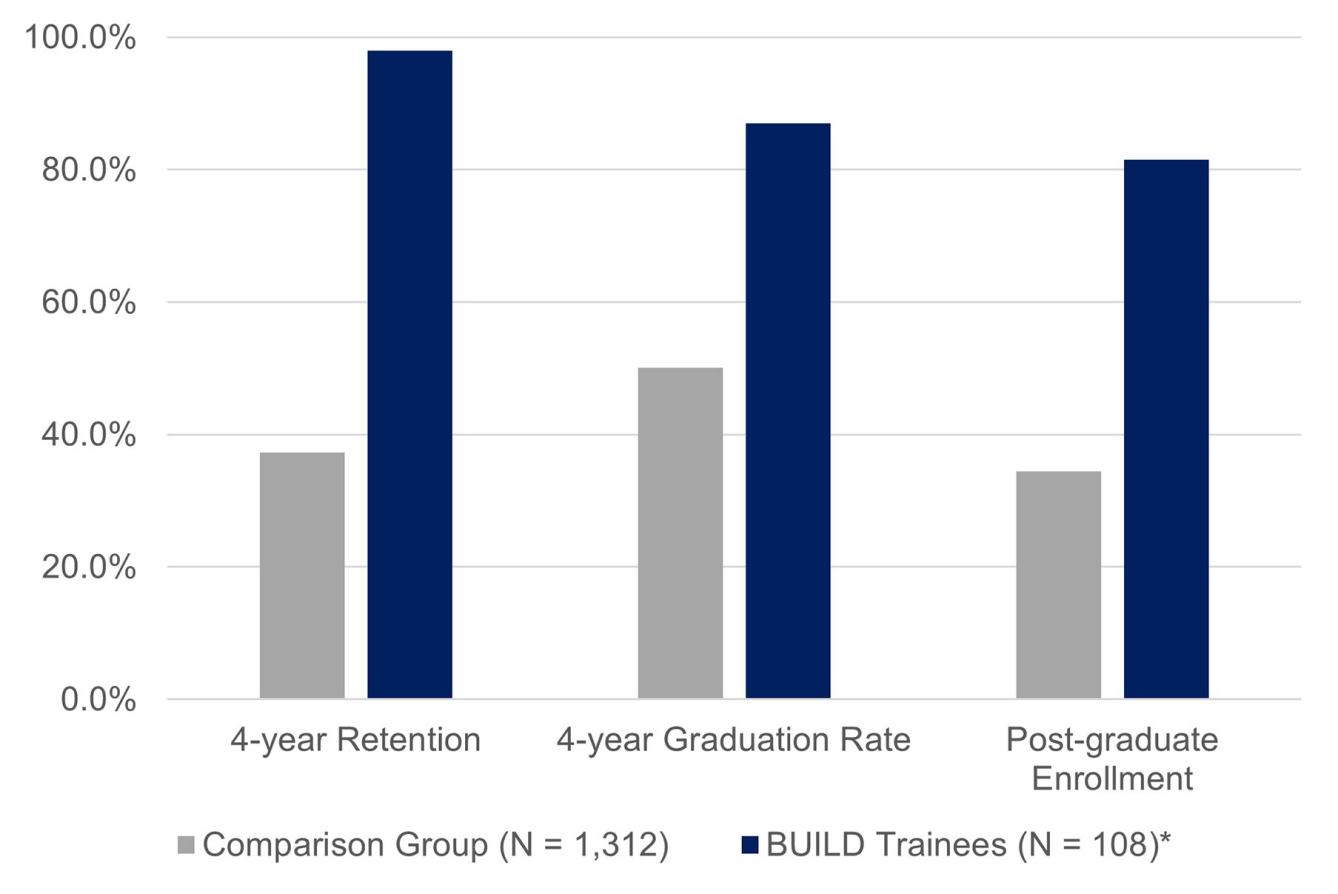
Academic metrics for the combined 2015, 2016 and 2017 program cohorts and comparison group.

Academic metrics results for the program fellows and comparison group are presented in Table 7 and Fig 2.

**Table 7:** Comparison of academic metrics and relation to hallmarks.

	Avg. Cumulative GPA	4-year Retention	4-year Graduation Rate	Post-graduate Enrollment
**Comparison Group (N = 1,312)**	3.66	37.30%	50.10%	34.40%
BUILD Fellows (N = 108)*	3.68	98.00%	87.00%	81.50%
**Corresponding Hallmarks** (see Table 5)	STU-1 STU-12	STU-9 STU-12 STU-16	STU-1 STU-12	STU-17 STU-18

*Includes all three cohorts (15–16, 16–17, and 17–18).

These results show that, after accounting for retention, 4-year graduation and post-graduate enrollment, out of 108 program participants in the three cohorts, 74 accomplished the objective of the program of entering graduate or advanced professional programs, which corresponds to a 69% success rate. For the comparison group, however, 83 out of 1,312 students entered post-graduate school, which corresponds to 6.3%.

Multiple questions arise when looking at these results. For example, why is the 4-year retention of the comparison group so low in contrast with that of program participants (60 percentage points difference)? What factors contribute to students in the program being highly retained while those in the comparison group being pushed out? A study conducted by Talaifar et.al. [31] suggests that developing identity fusion (oneness with a group) within the university is significantly related to student retention, even when controlled for demographics, cumulative GPA, or personality. That study showed that although grades did provide a reliable pathway to retention, the effect of identity fusion followed an independent pathway to retention, concluding that students fused with a university integrate academic life into their emerging sense of self and long-term identities. This is clearly a factor influencing the retention of students in the BUILDing SCHOLARS program, as our efforts include the development of a cohesive community of practice with common intellectual experiences through multiple social and academic/research activities. These factors were individually mentioned by some program alumni during interviews conducted for a recent qualitative study [32]. In that study, several students who had already graduated from the program reported local and external summer research experiences at partner institutions as important factors in their desire to continue with the program (identity fusion) and pursue advance studies. Many also indicated that material resources (tuition scholarship, stipend, travel to summer programs and conferences) were critical factors in their commitment to the training provided by the program. Leone et al. [33], describes five push versus pull forces that influence student retention: financial aid package, degree programs, campus life, campus location and food services. Given that UTEP’s student population is majority commuter (local–live with family) and financially disadvantaged, and that the comparison group had a similarly high GPA at the end of their first year, we suspect that lack of finances and inability to enjoy life on campus are the most influencing forces pushing comparison group students out, with the consequential inability to develop identity fusion. However, there are other factors known to push STEM students out, which may also be at play within the comparison group. For example, STEM subjects can be demanding, requiring strong analytical and problem-solving skills. As students move to their sophomore year and beyond, if they find the coursework too challenging, they may feel discouraged and decide to switch to less demanding fields [34]. Another push factor may be a perceived lack of support, as students who are not part of a mentored cohort might feel inadequately supported by professors, advisors, and/or university resources, leading to feelings of isolation and frustration [35–37]. The perception of limited employment opportunities in STEM fields, due to a lack of information about career prospects in the field of their chosen major may also be pushing non-program students out, leading them to pursue other majors that seem more promising in terms of employment. This factor may be augmented by family pressure, particularly for students who are financially disadvantaged and first in their families to go to college [37,38]. Finally, if relevance to their lives or communities is lacking and students do not see how STEM knowledge can be applied in real-world scenarios, they may lose interest in continuing their studies in these fields [39].

Another interesting comparison is the 4-year graduation rate. For the program fellows who were retained, the 4-year graduation rate is not 100%. Looking deeper into the records of program fellows who took longer than 4 years to graduate, we discovered that, since the program aims to prepare biomedical researchers, participants with engineering majors were required to minor in biomedical engineering to qualify for the program. Consequently, those students took one extra year to graduate, as in addition to the minor, their engineering degree requires a few additional credit hours. Nevertheless, there is a significant 37-percentage point difference between retained program participants and retained students in the comparison group. Why are the students in the comparison group who were retained not graduating within four years? What are the specific factors slowing them down? Witteveen et al. [40], identified five “structural forces” that increase time-to-degree: part-time enrollment, remedial coursework, transfers between institutions, stopping out, and changing or double majors. Of these forces, given these students’ high GPA, part-time enrollment due to financial constraints and work obligations appear to be the predominant forces for our comparison group.

Furthermore, to address post-graduate enrollment for program participants versus the comparison group, we determined the number of students in each group who enrolled in a degree post-graduation out of those who graduated in 4 years. The difference between program participants and the comparison group is larger than their differences in other metrics: 47 percentage points. A word of caution is in order here. We stay in touch with most program participants after they graduate, which we cannot do with students in the comparison group. Therefore, we can easily update program participants’ post-graduation status, which has resulted in a 10-percentage point increase from the information provided by the Student Clearinghouse data. However, even when considering this potential error (numbers may be higher for the comparison group as well), the difference is still substantially large. Are there specific aspects of the program that influence more students to apply and enroll? This is a prime area for a qualitative study. However, we attribute the difference to three factors: first, the program sets up high expectations, emphasized from the time students are recruited into the program all the way to their graduation. Second, adding to the program’s high expectations, is the strong encouragement from faculty, post-doctoral fellows and graduate students in the research teams where students conduct their projects. Third, as shown on Table 1 above, the program provided workshops for students to prepare their graduate school application materials, as well as for the GRE exam and their graduate school interview.

Each of the 108 students presented their research projects at least five times. Depending on their entry point into research, the project conducted with their UTEP faculty mentor was presented at least twice at a local, campus-wide symposium. Also depending on their entry point into the program, the projects conducted with summer research mentors at partner institutions were presented at least twice at a formal local symposium organized by the program upon their return from the experience. All students were also provided with funds to present once a year at a national conference. Topics of student thesis projects fell under one of the research nodes described above. Table 8 lists the number of student projects in each of the nodes, including projects that were transdisciplinary.

**Table 8:** Student thesis topic per node.

Research Node	Student Thesis Topic
Addiction	11
Cancer	13
Environmental Health	3
Health Disparities	18
Infectious Diseases	16
Neurodegenerative Diseases	8
Translational Biomedicine	6
Transdisciplinary	31
**Total**	**108**

Finally, there were 14 student publications in refereed journals from the three cohorts reported here. Nine of these were with their UTEP academic year mentors and five with their summer research mentors at partner institutions. All publications were reported in and tracked through the NIH Manuscript Submission System (NIHMS) [41], as well as the program database. While most of the articles were published before the students graduated, some have been published after their graduation.

### 4.2 Non-academic metrics: research self-efficacy, science identity and quality of research experience

#### 4.2.1. Research self-efficacy

**Fig 3:**
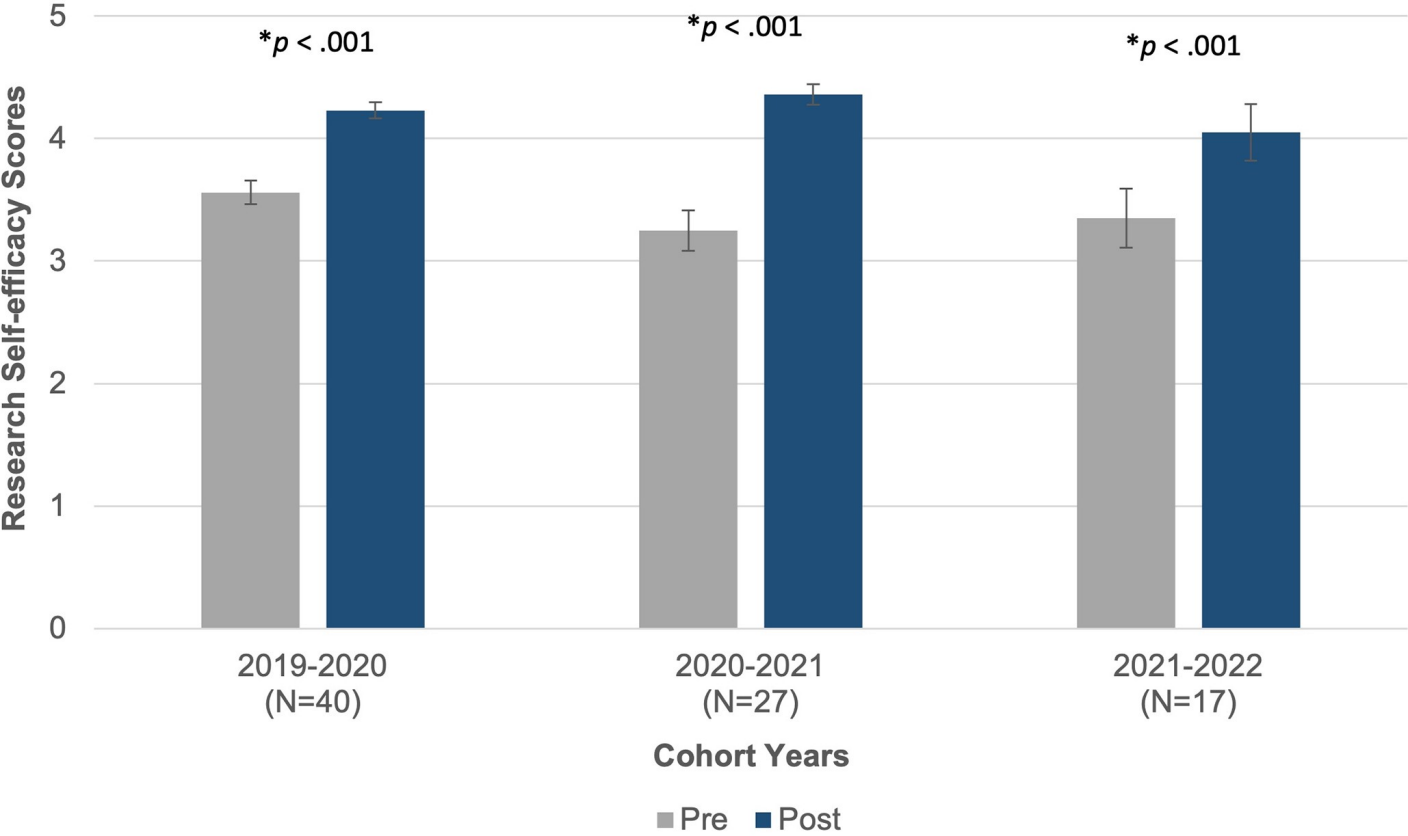
Cross sectional pre- and post- research self-efficacy scores.

We used IBM SPSS Statistics (Version 29) to conduct two-tailed paired samples t-tests with each pair of retrospective pre- and post-research self-efficacy scores by survey: 2019–2020, 2020–2021, and 2021–2022. Each analysis provided a positive and significant increase in the program’s fellows’ research self-efficacy. For the 2019–20 cohort, the program fellows (*n* = 40) showed a significant increase from before their 2019–2020 research experience (*M* = 3.56, *SD* = 0.61) to after their research experience (*M* = 4.23, *SD* = 0.42; *t*(39) = 8.49, *p* < .001). The program fellows who completed the 2020–2021 survey (*n* = 27) also indicated a significant increase from before their academic year research experience (*M* = 3.25, *SD* = 0.86) to after their research experience (*M* = 4.36, *SD* = 0.44; *t*(27) = 7.10, *p* < .001). Finally, the program fellows (*n* = 17) in the 2021–2022 survey indicated significantly greater research self-efficacy scores after their research experience (*M* = 4.05, *SD* = 0.95) compared to before their research experience (*M* = 3.35, *SD* = 0.99; *t*(16) = 4.71, *p* < .001). See Fig 3.

#### 4.2.2 Science identity

**Fig 4:**
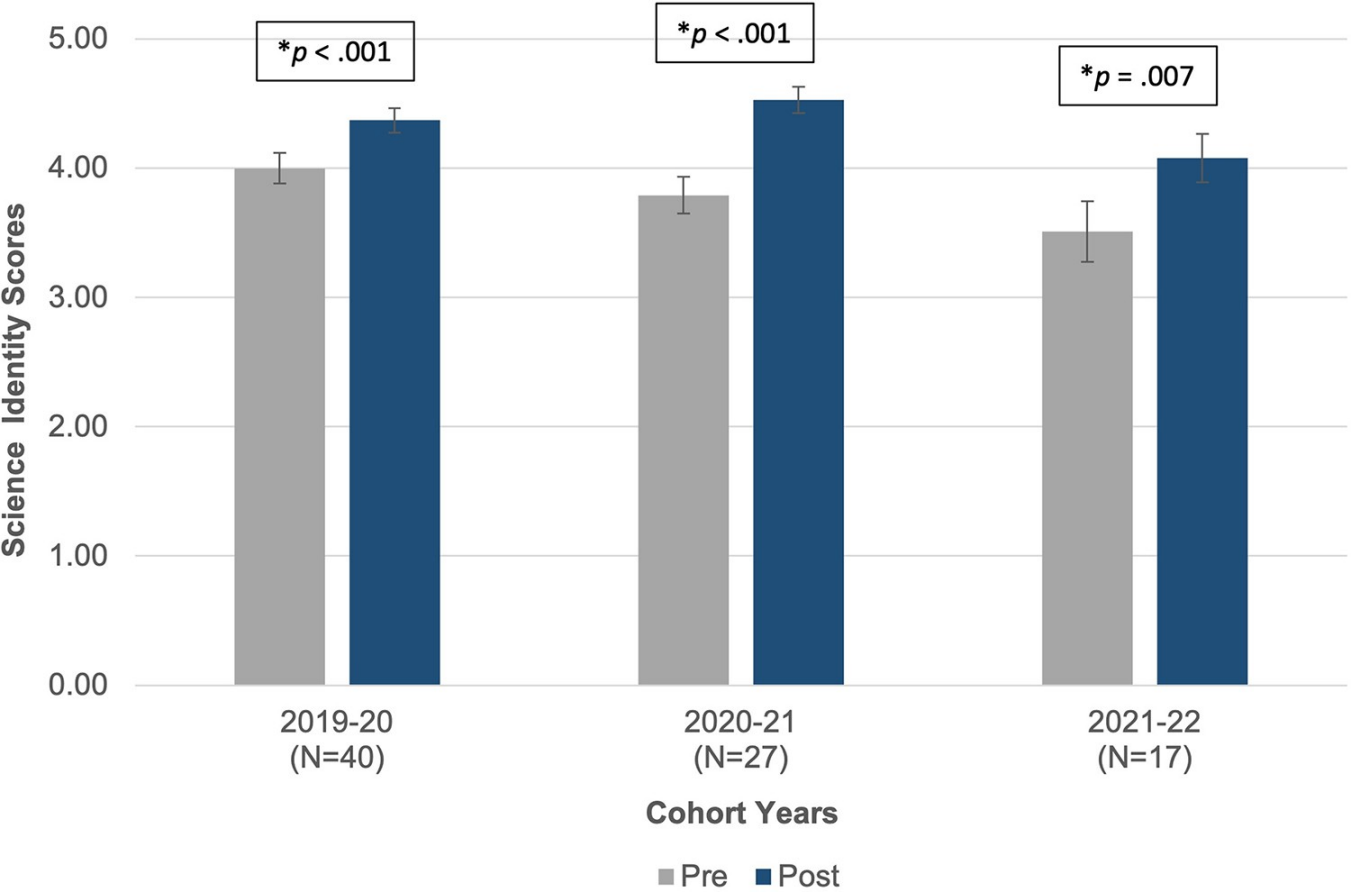
Cross-sectional pre- and post-science identity scores.

Overall, the analysis indicates that the program fellows’ science identities increased as a result of their academic year research experiences. The fellows who completed the survey in the 2019–2020 (*n* = 40) academic year have a lower science identity before (*M* = 4.00, *SD* = 0.76) than after their research experience (*M* = 4.37, *SD* = 0.61; *t*(39) = 5.19, *p* < .001). For the 2020–2021 academic year, the program fellows (*n* = 27) similarly showed a lower science identity before their research experience (*M* = 3.79, *SD* = 0.74) than after (*M* = 4.53, *SD* = 0.64; *t* (26) = 5.84, *p* < .001). Finally, the fellows (*n* = 17) reported similar gains in their science identity during their 2021–2022 mentored research experience, indicating a lower science identity before their experience (*M* = 3.51, *SD* = 0.96) compared to after their research experience (*M* = 4.08, *SD* = 0.77; *t*(16) = 3.08, *p* = .007). Fig 4 compiles these four cross-sectional analyses into one graph.

#### 4.2.3 Quality of the research experience

As indicated in the previous section, students self-reported their increase in research self-efficacy using a retrospective pre-post survey. Results on the quality of the fellows’ research experience using the MCA surveys is being published separately. Likewise, the role of research mentoring on the quality of fellows’ research presentations as well as faculty barriers and benefits in mentoring BUILDing SCHOLARS in research have already been addressed in prior publications [30,42].

Lastly, interviews conducted for a qualitative study to understand how fellows utilized programmatic experiences when applying to graduate school showed that support from their research mentors and peers was one of the primary sources of cultural capital. It also showed how they used the community cultural wealth they acquired during their participation in the program to challenge feeling like an outsider in their graduate environment [32].

## 5. Conclusion

The body of research on STEM recruitment, retention, and inclusion supports activities associated with advising and mentoring, early research experiences, network development, family engagement and curricular reforms involving inclusive and supportive learning environments. The BUILDing SCHOLARS student development program implemented all these activities, leading to extremely positive outcomes on retention (98.0%), development of research self-efficacy and science identity, 4-year graduation rate for those retained (87.0%), and entry into advanced degrees for those who graduated in 4 years (81.5%), thus supporting the importance of using an asset bundles approach to training underrepresented students to pursue advanced studies and careers in biomedical areas. Future research should explore the long-term career outcomes of program participants and the potential for expanding these initiatives to other STEM fields. In this vein, we will continue using program data to conduct rigorous analysis on more specific factors that increase the academic and non-academic outcomes of biomedical research trainees. We acknowledge that issues surrounding scalability of the program components and the efficacy of replicating the model in other contexts is a highly salient issue. Future research should use multi-institutional data to investigate how specific factors, shown to be impactful in one context, can translate to other institutions. By combining institutional level data with program level data, insights about replicating interventions to other settings could inform a broader range of institutions looking to improve biomedical research training programs. Moreover, policy papers could provide guidance about the best practices for scaling up proven interventions. Collectively, these further studies will provide further insights into how interventions in research training function within particular institutional contexts and disciplines.

Institutions interested in improving retention rates of minority students in STEM, their time to degree, graduation rates and their entry into advanced degree programs should look into factors such as financial aid and scholarships, extended research mentoring, cohort building, professional development training, family engagement and STEM career advice to provide a higher level of inclusion, which lead to better preparation, as well as increases in science identity and sense of belonging in STEM. Providing financial aid and scholarships is central to alleviating economic barriers and enabling students to focus on their studies. Multiple research experiences and travel to conferences lead to the development of networks that provide a sense of confidence when it is time to apply to advanced degree programs, especially for students who are first generation and have only left home to attend summer research programs at other institutions. Extended research experiences at the home institution often results in substantial productivity in the form of an undergraduate thesis and publications, adding to student growth in science identity and research self-efficacy. Moreover, extended and multiple research experiences include regular interactions with faculty and peer mentors, which also play a significant role in fostering academic and professional growth. Professional development training equips students with the skills necessary for successful careers, while family engagement initiatives ensure that students receive support from their home environments. Additionally, offering STEM career advice helps students navigate and reflect on their educational and professional journeys, leading to better preparation, increased science identity, and a stronger sense of belonging in STEM fields. Finally, inclusion of fellows’ family through family engagement activities, such as workshops and informational sessions, empower families to not only support their students effectively, but also to better understand the strong contributions of researchers to advancements in medicine and health maintenance.

Provided the evidence presented about the impact and efficacy of this undergraduate biomedical research training program, it is noteworthy that efforts are underway at the institution to ensure that the most impactful elements of the program are sustainable in the long-term. In particular, the university is supporting a 60% tuition scholarship for 18 students per academic year and research partner institutions will continue to provide pathways for students from the institution to apply to their funded summer research programs. This last step of ensuring sustainability of core program components is crucial so that students continue to receive support as they pursue biomedical research careers.

## Supporting information

S1 Table Supplementary information for Table 6 BUILD fellows. Academic metrics for program fellows.
(XLSX)

S2 Table Supplementary information for Table 6 comparison group. Academic metrics for comparison group.
(XLSX)

S1 File RSE & SI scales. Research Self-efficacy and Science Identity Scales.
(DOCX)

S2 File Figs 3 & 4. Research Self-efficacy and Science Identity.
(XLSX)

S3 File Figs 3 & 4. Research Self-efficacy and Science Identity raw data with merged values.
(XLSX)

S4 File Figs 3 & 4. Research Self-efficacy and Science Identity SPSS output.
(DOC)
