# Supplementary material for: BUILDing SCHOLARS: A program exemplar at a Hispanic serving institution to develop biomedical researchers
Source: PLoS One. 2024 Dec 30;19(12):e0315298. doi: 10.1371/journal.pone.0315298 (PMC11684598; doi:10.1371/journal.pone.0315298)
Supplement: S1 File — Research Self-efficacy and Science Identity Scales. (DOCX) [file pone.0315298.s003.docx]

Science Identity

To what extent do you disagree or agree that the following statements were/are (***before*** and ***after*** **this Academic Year’s RESEARCH experience**) true of you:

|  | *Before* this Academic Year’s RESEARCH Experience | | | | | *After* the Academic Year’s RESEARCH Experience | | | | |
| --- | --- | --- | --- | --- | --- | --- | --- | --- | --- | --- |
|  | Strongly Disagree (1) | Disagree (2) | Neither Agree nor Disagree (3) | Agree (4) | Strongly Agree (5) | Strongly Disagree (1) | Disagree (2) | Neither Agree nor Disagree (3) | Agree (4) | Strongly Agree (5) |
| I have a strong sense of belonging to the community of scientists |  |  |  |  |  |  |  |  |  |  |
| I derive great personal satisfaction from working on a team that is doing important research |  |  |  |  |  |  |  |  |  |  |
| I have come to think of myself as a ‘scientist’ |  |  |  |  |  |  |  |  |  |  |
| I feel like I belong in the field of science |  |  |  |  |  |  |  |  |  |  |

Research self-efficacy

***Based on this Academic Year’s research experience***, please use the scale below and indicate how much you disagree or agree that you had/have the confidence in your ability to do each task/activity at the beginning of the experience and also now at the end.

|  | At the ***beginning of the Research experience*** | | | | | Now at the **end of this year’s Research experience** | | | | |
| --- | --- | --- | --- | --- | --- | --- | --- | --- | --- | --- |
|  | Disagree Completely (1) | Disagree (2) | Undecided (3) | Agree (4) | Agree Completely (5) | Disagree Completely (1) | Disagree (2) | Undecided (3) | Agree (4) | Agree Completely (5) |
| I can realize the problems that may contribute to the field I work in. |  |  |  |  |  |  |  |  |  |  |
| I believe I am sufficient in creating hypotheses relevant to my research. |  |  |  |  |  |  |  |  |  |  |
| I can explain my research problem by drawing the necessary connections with prior research results. |  |  |  |  |  |  |  |  |  |  |
| I can find an appropriate title to my research. |  |  |  |  |  |  |  |  |  |  |
| I can effectively carry out a literature search by using various tools (internet, library, etc). |  |  |  |  |  |  |  |  |  |  |
| I systematically keep record of the results of the literature search. |  |  |  |  |  |  |  |  |  |  |
| I do not find it difficult at all to compare the results of my research to prior research results. |  |  |  |  |  |  |  |  |  |  |
| I can criticize the results of my research regarding research processes. |  |  |  |  |  |  |  |  |  |  |
| I can define the appropriate sampling method for my research. |  |  |  |  |  |  |  |  |  |  |
| I can decide which approaches to use for my research problem (quantitative, qualitative, various approaches, etc.). |  |  |  |  |  |  |  |  |  |  |
| I can choose the appropriate data collection method for my research. |  |  |  |  |  |  |  |  |  |  |
| I can test the validity and reliability of my research data through appropriate methods. |  |  |  |  |  |  |  |  |  |  |
| I can choose appropriate statistical methods to test or respond to my research hypotheses. |  |  |  |  |  |  |  |  |  |  |
| I can appropriately report on my analysis results. |  |  |  |  |  |  |  |  |  |  |
| I can discuss my research findings within a conceptual framework. |  |  |  |  |  |  |  |  |  |  |
| I can create an appropriate category system when writing up my research. |  |  |  |  |  |  |  |  |  |  |
| I can utilize appropriate referencing in my research, whether direct or indirect. |  |  |  |  |  |  |  |  |  |  |
| I can write an abstract to my research with ease. |  |  |  |  |  |  |  |  |  |  |

Büyükoztürk, S., Atalay, K., Sozgun, Z., & Kebapcı, S. (2011). The development of research self-efficacy scale. *Cypriot Journal of Educational Sciences*, *6*(1), 22-29.

Chemers, M. M., Zurbriggen, E. L., Syed, M., Goza, B. K., & Bearman, S. (2011). The role of efficacy and identity in science career commitment among underrepresented minority students. *Journal of Social Issues*, *67*(3), 469-491.

Estrada, M., Woodcock, A., Hernandez, P. R., & Schultz, P. W. (2011). Toward a model of social influence that explains minority student integration into the scientific community. *Journal of educational Psychology*, *103*(1), 206-222.
