# Supplementary material for: BUILDing SCHOLARS: A program exemplar at a Hispanic serving institution to develop biomedical researchers
Source: PLoS One. 2024 Dec 30;19(12):e0315298. doi: 10.1371/journal.pone.0315298 (PMC11684598; doi:10.1371/journal.pone.0315298)
Supplement: S4 File — Research Self-efficacy and Science Identity SPSS output. (DOC) [file pone.0315298.s006.DOC]

**BUILD 2019-2020** (taken from file 2020-2021)

| **Paired Samples Statistics** | | | | | |
| --- | --- | --- | --- | --- | --- |
|  | | Mean | N | Std. Deviation | Std. Error Mean |
| Pair 1 | Research Self-Efficacy After | 4.2307 | 40 | .41953 | .06633 |
|  | Research Self-Efficacy Befor | 3.5595 | 40 | .60752 | .09606 |
| Pair 2 | Science Identity After | 4.3688 | 40 | .60708 | .09599 |
|  | Science Identity Before | 4.0000 | 40 | .75955 | .12010 |

| **Paired Samples Test** | | | | | | | | | |
| --- | --- | --- | --- | --- | --- | --- | --- | --- | --- |
|  | | Paired Differences | | | | | t | df | Sig. (2-tailed) |
|  |  | Mean | Std. Deviation | Std. Error Mean | 95% Confidence Interval of the Difference | |  |  |  |
|  |  |  |  |  | Lower | Upper |  |  |  |
| Pair 1 | Research Self-Efficacy | .67125 | .49985 | .07903 | .51139 | .83111 | 8.493 | 39 | .000 |
| Pair 2 | Science Identity | .36875 | .44931 | .07104 | .22505 | .51245 | 5.191 | 39 | .000 |

**BUILD 2020 – 2021 (**taken from BUILD 21-22**)**

| **Paired Samples Statistics** | | | | | |
| --- | --- | --- | --- | --- | --- |
|  | | Mean | N | Std. Deviation | Std. Error Mean |
| Pair 1 | Research Self Efficacy Post- | 4.3574 | 27 | .43865 | .08442 |
|  | Research Self Efficacy Pre- | 3.2512 | 27 | .83228 | .16017 |
| Pair 2 | Science Identity Post-Comp | 4.5278 | 27 | .64425 | .12399 |
|  | Science Identity Pre-Comp | 3.7870 | 27 | .73936 | .14229 |

| **Paired Samples Test** | | | | | | | | | |
| --- | --- | --- | --- | --- | --- | --- | --- | --- | --- |
|  | | Paired Differences | | | | | t | df | Sig. (2-tailed) |
|  |  | Mean | Std. Deviation | Std. Error Mean | 95% Confidence Interval of the Difference | |  |  |  |
|  |  |  |  |  | Lower | Upper |  |  |  |
| Pair 1 | Research Self Efficacy | 1.10622 | .80930 | .15575 | .78608 | 1.42637 | 7.103 | 26 | .000 |
| Pair 2 | Science Identity | .74074 | .65955 | .12693 | .47983 | 1.00165 | 5.836 | 26 | .000 |

**BUILD 2021 – 2022**

| **Paired Samples Statistics** | | | | | |
| --- | --- | --- | --- | --- | --- |
|  | | Mean | N | Std. Deviation | Std. Error Mean |
| Pair 1 | r_se_post | 4.0523 | 17 | .95429 | .23145 |
|  | r_se_pre | 3.3497 | 17 | .99260 | .24074 |
| Pair 2 | sci_id_post | 4.0784 | 17 | .77419 | .18777 |
|  | sci_id_pre | 3.5147 | 17 | .95799 | .23235 |

| **Paired Samples Test** | | | | | | | | | |
| --- | --- | --- | --- | --- | --- | --- | --- | --- | --- |
|  | | Paired Differences | | | | | t | df | Sig. (2-tailed) |
|  |  | Mean | Std. Deviation | Std. Error Mean | 95% Confidence Interval of the Difference | |  |  |  |
|  |  |  |  |  | Lower | Upper |  |  |  |
| Pair 1 | r_se_post - r_se_pre | .70261 | .61518 | .14920 | .38632 | 1.01891 | 4.709 | 16 | .000 |
| Pair 2 | sci_id_post - sci_id_pre | .56373 | .75434 | .18296 | .17588 | .95157 | 3.081 | 16 | .007 |
